# UPLC-qTOF-MS phytochemical profile of *Commiphora gileadensis* leaf extract via integrated ultrasonic-microwave-assisted technique and synthesis of silver nanoparticles for enhanced antibacterial properties

**DOI:** 10.1016/j.ultsonch.2024.106923

**Published:** 2024-05-25

**Authors:** Hani Ahmed, Mohamed Y. Zaky, Marwan M. A. Rashed, Marwan Almoiliqy, Sam Al-Dalali, Zienab E. Eldin, Mohanad Bashari, Ahmad Cheikhyoussef, Sulaiman A. Alsalamah, Mohammed Ibrahim Alghonaim, Abdulrahman M Alhudhaibi, Jinpeng Wang, Li-Ping Jiang

**Affiliations:** a School of Pharmaceutical Science, Nanchang University, Nanchang 330006, Jiangxi, China; b Molecular Physiology Division, Faculty of Science, Beni-Suef University, Egypt; c School of Biological and Food Engineering, Suzhou University, Suzhou 234000, Anhui, China; d Department of Medicine and Health Science, College of Medicine and Health Science, University of Science and Technology, Aden, Yemen; e Department of Food Science and Technology, Ibb University, Ibb 70270, Yemen; f Department of Food Science and Human Nutrition, College of Applied and Health Sciences, A’Sharqiyah University, Ibra, Oman; g Science and Technology Division, Multidisciplinary Research Centre, University of Namibia, Windhoek, Namibia; h Department of Biology, College of Science, Imam Mohammad Ibn Saud Islamic University, Riyadh 11623, Saudi Arabia; i Key Laboratory of Geriatric Nutrition and Health (Beijing Technology and Business University), Ministry of Education, Beijing, China; j School of Food and Health, Beijing Technology and Business University, Beijing, China

**Keywords:** *Commiphora gileadensis*, Ultrasonic-microwave-assisted, Green silver nanoparticles, Metabolite profiling, UPLC-qTOF-MS, Antimicrobial

## Abstract

The utilization of metallic nanoparticles in bio-nanofabrication holds significant potential in the field of applied research. The current study applied and compared integrated ultrasonic-microwave-assisted extraction (US/MICE), ultrasonic extraction (USE), microwave-assisted extraction (MICE), and maceration (MAE) to extract total phenolic content (TPC). In addition, the study examined the antioxidant activity of *Commiphora gileadensis* (*Cg*) leaf. The results demonstrated that the TPC of US/MICE exhibited the maximum value at 59.34 ± 0.007 mg GAE/g DM. Furthermore, at a concentration of 10 μg/mL, TPC displayed a significant scavenging effect on DPPH (56.69 %), with an EC_50_ (6.48 μg/mL). Comprehensive metabolite profiling of the extract using UPLC-qTOF-MS/MS was performed to identify active agents. A total of 64 chromatographic peaks were found, out of which 60 were annotated. The most prevalent classes of metabolites found were polyphenols (including flavonoids and lignans), organic compounds and their derivatives, amides and amines, terpenes, and fatty acid derivatives. Transmission electron microscopy (TEM) revealed the aggregate size of the synthesized nanoparticles and the spherical shape of *C. gileadensis*-mediated silver nanoparticles (*Cg*-AgNPs). The nanoparticles had a particle size ranging from 7.7 to 42.9 nm. The *Cg*-AgNPs exhibited more inhibition zones against *S. aureus* and *E. coli*. The minimum inhibitory concentration (MIC) and minimum bactericidal concentration (MBC) of *Cg*-extract, AgNPs, and *Cg*-AgNPs were also tested. This study demonstrated the feasibility of using combined ultrasonic-microwave-assisted extraction to separate and extract chemicals from *C. gileadensis* on a large scale. These compounds have potential use in the pharmaceutical industry. Combining antibacterial and biocompatible properties in materials is vital for designing new materials for biomedical applications. Additionally, the results showed that the biocompatibility of the Ag-NPs using *C. gileadensis* extracts demonstrated outstanding antibacterial properties.

## 1 Introduction

Recently, due to the possible toxicity of synthetic antioxidants and the rise of antibiotic resistance, numerous botanical extracts have garnered significant interest as natural sources of antioxidants and antimicrobial agents. Nanotechnology is a crucial and viable method for maintaining plant-based biologically active compounds [1], [2].

Nanoparticles (NPs) play essential roles in biotechnology and pharmaceutical applications, making them indispensable [3]. The chemical, biological, and physical characterization of produced nanoparticles is a fast-expanding area of research. There have been extensive studies on the biosynthesis of nanoparticles of plant extracts [4]. These nanoparticles have been found to possess antimicrobial agents [5], [6], antioxidants, and anti-glycation agents [7]. In addition, there has been a growing interest in using silver nanoparticles (AgNPs) because they are environmentally friendly, cost-effective, and safe [8]. For instance, silver has been utilized in medicine for an extensive period [3].

Furthermore, AgNPs exhibited antibacterial, anticancer, antifungal, and enzyme mimic properties [9]. Various factors can affect the NP properties, such as experimental conditions, the relationship of interactions between metal ions and reducing substances, and the preparation method. Nanoparticles are strongly impacted by size, shape, and distribution, often controlled by changing synthetic techniques and reducing agents and stabilizers [10]. Recent research trends have indicated that various plant components, including extracts from *Origanum vulgare* [11], *Piper nigrum* seeds extract [12], *Tropaeolum majus* leaves [13], *Ribes nigrum* fruit [14], and *Cyperus conglomeratus* roots [15]. Numerous plant extracts have attracted significant interest as natural sources of antioxidants and antimicrobial agents due to synthetic antioxidants potential toxicity and antibiotic resistance [16], [17]. Plant-sourced phenolic compounds (PSPC) are abundant in spices, seeds, vegetables, fruits, and aquatic plants. The PSPC has played a critical role in scientific research and investigation of the pharmaceutical and cosmetic industries due to its remarkable antioxidant activity [18].

Several emerging technologies, including ultrasound and microwave radiation, are efficiently utilized to improve the extraction of plant-sources bioactive compounds [19], [20]. The collapse phenomenon of the cavitation bubble caused by shock-controlled acoustic ultrasonic may intensify mass transfer, facilitating solvent flow into plant tissues. Consequently, the extraction of phenolic compounds from natural sources is improved [21]. Furthermore, these techniques have the potential to utilize the physical and chemical phenomena occurring within plant cells, which primarily distinguish them from traditional extraction techniques such as maceration and aqueous distillation. Consequently, ultrasonic technology can increase extraction performance, reduce chemical hazards, and accelerate the processing time [19].

*Commiphora gileadensis (Cg)* is a Burseraceae family member that can grow as a small tree. Southern Arabia and Northeastern Africa are two of its primary growing regions [22]. In Yemen and southern Saudi Arabia, it is referred to as Bisham. Due to their anti-inflammatory and analgesic properties, theses plants are utilized extensively in traditional medicine to treat various diseases. Moreover, the oil is associated with a multitude of health benefits. It is used to cure cold phlegm disorders such as epilepsy, paralysis, tetanus, and gonorrhea. Its oil is also used to massage arthritic joints by mixing it with other oils. The plant contains chlorophyll, carotenoids, and lycopene in the leaf extract, while a high level of proanthocyanidins was found in the stem peel extract [23]. A study conducted on the *C. gileadensis* plant demonstrated that employing modern techniques yields an extract exhibiting elevated activity in its biological properties [24].

The current study aims to assess the antibacterial activity of green-synthesized *Cg*-AgNPs against certain pathogenic bacteria. This was done using the disk diffusion method, minimum inhibitory concentration (MIC), and minimum bactericidal concentration (MBC) assays. In addition, the study assessed the efficacy of novel strategies using ultrasonic-homogenizer (US), microwave (MIC), and integrated ultrasonic-microwave (US/MIC) techniques compared with the conventional method. The study also measured the total phenolic content (TPC) and antioxidant activity of *C. gileadensis*. To our knowledge, this is the first study to examine the leaf extract of *C. gileadensis* synthesized using the integrated ultrasonic-microwave (US/MIC) technique and assess the antibacterial activity of *Cg*-AgNPs.

## 2 Materials and methods

### 2.1 Plant sample

Leaves of *C. gileadensis* were collected from Lawdar village, south-western Yemen, between March and September 2021. The geographic coordinates are longitude (45° 51′ 53″ E), and latitude (13° 53′ 11″ N). This place is 3254 ft above sea level. The *C. gileadensis* leaves were dried at 25 °C for ten days in a shaded spot and ground using an electric grinder. The acceptable eligible powder samples were kept in self-sealing polyethylene plastic bags at 4 °C.

### 2.2 Ultrasonic-homogenizer

The JY98-III DN Ultrasonic-homogenizer (ultrasonic power 1200 W at 20 kHz) utilized a jacketed beaker with a volume of 100 mL. It also included a digital LCD, a thermometer, and a water bath. It was manufactured by Nanjing FeiQi Industry & Trade Co., Ltd.

located in Nanjing, China.

### 2.3 Cooperative ultrasonic-microwave extractor-reactor (CW-2000)

The Ultrasonic/Microwave Cooperative Extractor (CW-2000) (Shanxi, Xi'an, China) with a 4″ LCD digital display was used for the integrated techno-extraction approaches. The apparatus operated under the following conditions: a microwave power range of 10–800 W, a frequency of 2,450 MHz, a fixed ultrasonic power level of 50 W, and an ultrasonic frequency of 40 kHz. A 500-mL reactor, which had the capacity to hold the sample within its 500-milliliter, was utilized in the experiment.

### 2.4 Chemicals

Glucose, Galactose, Agar, yeast, and Peptone were purchased from Hangzhou Haotian Biotechnology Co., Ltd (China). Silver nitrate (AgNO_3_) was purchased from Tianjin Dongjulong Chemical Technology Co. Ltd (China). Hard-Plus Resin-812 (GP18010), Uranyl acetate (GZ02616), and Lead citrate (GZ02616) were acquired from Beijing Zhongjingkeyi Technology Co., Ltd (ZJKY) China. Osmium tetroxide (GP18456) was sourced from Leica, (Germany). Sucrose (G8270) was provided by Sigma-Aldrich Corp., USA. PBS Buffer (YM-XZ002) was procured from Shanghai Yuanmu Biotech (China). Folin-Ciocalteu reagent and Gallic acid were purchased from Sigma-Aldrich, Germany. DPPH, ABTS, and TBHQ were sourced from TCI EUROPE N.V., Belgium. Trolox was purchased from Sigma-Aldrich USA.

### 2.5 Extraction of *Cg*-polyphenols

Different techniques were utilized to extract the *Cg*-polyphenols, including the maceration extract (MACE), ultrasonic-homogenizer assisted extraction (USE), ultrasonic-microwave assisted extraction (US/MICE) [25], and microwave-assisted extraction (MICE) [26]. All extracted samples were stored at a temperature of −4 °C until further analysis. The extraction yield (%) was determined based on the following equation:

(1) Yield%=(Weightofdriedextract/Weightofsample)×100

### 2.6 Analysis of *cg*-polyphenol extracts

#### 2.6.1 Determination of total phenolic content (TPC)

The TPC of *C. gileadensis* powder extract was determined using Singleton and Rossi's Colorimetric Oxidation Reaction technique, as described by Rashed et al. [25]. The results were presented as mg gallic acid equivalents per gram of dry matter (mg GAE/g DM), using the standard curve of Gallic acid (*R^2^ = 0.99*).

#### 2.6.2 Determination of antioxidant activity using the DPPH and ABTS methods

The antioxidant scavenging effect of *C. gileadensis* was assessed based on the DPPH assay [25] and ABTS assay [27], according to Rashed et al., without any modifications. The results were expressed using the following equation:

(2) DPPH%=[(A-B)/A]×100

where: A is the optical density of the control sample, and B is the optical density of the sample. TBHQ served as a positive sample at 120 mg/L. There were three duplicates of each treatment.

The inhibition effect for the ABTS assay was quantified in terms of mM Trolox equivalents (*R^2^* = 0.9965).

### 2.7 UHPLC‐Qtof‐MS/MS analysis

The sample analysis used a Hybrid Quadrupole-TOF Mass Spectrometer Triple TOF (AB Sciex). An LC30 system (Shimadzu, Japan) with a Hybrid Quadrupole-TOF Mass Spectrometer: Triple TOF 5600 + by AB Sciex (USA) was used for chromatographic separation. The analyte was separated at 25 °C using a Shim-pack CIST C18 (2.1 mm × 75 mm, 2 μm) column. The mobile phase consisted of (A) acetonitrile (100 %) and (B) formic acid 0.1 % (v/v) in water. The settings of the mobile phase gradient ranged from 5 % B to 95 % B at a flow rate of 0.3 mL/min for 35 min.

The MS and MS/MS data were recorded using a Hybrid Triple TOF 5600 + system (AB Sciex) equipped with an electrospray ionization source (ESI). The MS and MS/MS data were collected using the following parameters: positive ionization mode, flow rate of 0.3 mL/min, spray gas pressure of 50 psi, mass range of *m*/*z* 70–1,100, capillary voltage of 3.5 kV, ISVF spray voltage of 5,500 V, ion source temperature of 550 °C. Helium and nitrogen were used for the collision gas and auxiliary, respectively. The Molecular Formula Calculator on Mass Hunter determined the elemental composition of each precursor and product ion.

The compounds' precise MS and MS/MS spectra determined by the QTOF mass analyzer served as the basis for the characterization approach. The chemical composition information and data were obtained using the literature from SciFinder Scholar (https://scifinder.cas.org), METLIN Metabolite Database (https://metlin.scripps.edu), and MassBank (https://massbank.jp).

### 2.8 Synthesis of AgNPs incorporated with extracts

The extract was prepared in order to synthesize AgNPs, following the procedure described in [28], [29]. A total of 80 mL of a 5 mM aqueous solution of AgNO3 was added to 20 mL of *C. gileadensis* extract that was prepared using ultrasonic-microwave (*Cg*-US/MICE). The resulting mixture was continuously stirred at 60 °C for 6 h until the solution changed from light brown to dark brown. Then, C*g*-AgNPs dispersion was centrifuged at 15,000 rpm for 15 min at 4 ℃ to eliminate the residual solution and obtain the precipitated nanoparticles. After C*g*-AgNPs were obtain, they were washed with deionized water and centrifuged at 10000 rpm for 5 min three times. Finally, the produced *Cg*-AgNPs were freeze-dried and kept at 4 °C until they were characterized using TEM.

### 2.9 Characterization of synthesized silver nanoparticles (AgNPs)

#### 2.9.1 X-ray diffraction (XRD)

The crystallinity of AgNPs was assessed by conducting X-ray diffraction (XRD) analysis using a Rigaku MiniFlex 600 diffractometer. The AgNP powder sample for XRD was prepared using drop-casting onto a glass slide and air-drying under ambient conditions. The XRD pattern was recorded using Cu Kα radiation (λ = 1.54 Å). The XRD diffractograms were obtained by scanning the powdered material in the 2θ range of 0–70° at a voltage of 40 kV and a current of 15 mA. A scanning range of 2θ/θ was selected, and a scanning speed of 2 min^−1^ was used.

#### 2.9.2 UV–Vis spectroscopy

The ultraviolet–visible (UV–vis) absorption spectrum of biosynthesized AgNPs was measured using a Shimadzu UV-3600 spectrophotometer (Japan). This instrument was used to record the samples' absorbance spectra of the samples in aqueous media at ambient temperature.

#### 2.9.3 Hydrodynamic size and Polydispersity Index (PDI)

The hydrodynamic size and Polydispersity Index (PDI) were determined using a ZS90 Zetasizer instrument (Malvern, UK). The analysis of AgNPs utilized Dynamic Light Scattering (DLS) as its underlying principle. The data obtained from DLS was subsequently analyzed using MalvernMalvern's 'DTS nano' software. All measurements were conducted in triplicate.

#### 2.9.4 Nanoparticles morphology (Transmission electron microscope (TEM) and scanning electron microscopy (SEM))

The morphology characteristics of AgNPs were analyzed using a JEM-1400PLUS TEM. Cg-AgNP samples were examined using TEM (JEM-1400PLUS, JEOL, Japan). First, the samples were pre-fixed with 3 % glutaraldehyde and subsequently fixed with 1 % tetroxy solution for 30 min. The dehydration process was conducted systematically using acetone, with concentration gradients of 30 %, 50 %, 70 %, 80 %, 90 %, 95 %, and 100 % (with three changes made at 100 % concentration). Following dehydration, the sample was embedded in epoxy resin (Epon 812) and a dehydrating agent in 3:1, 1:1, and 1:3 ratios, respectively. Each stage was processed for 30 to 60 min. The embedding solution obtained was poured into the mold that contained the infiltrated sample block. Following the application of heat and polymerization, a cohesive solid matrix, known as an embedding block, was generated, thereby facilitating the subsequent stages. Thin slices, approximately 50 nm thick, were prepared using a Leica EM UC7 ultra-thin microtome. These slices were then placed on the liquid surface of the cutter groove before being transferred onto the copper net. The samples were initially stained with uranyl acetate and then stained with PBD for 15–20 min at room temperature. The TEM analyses were conducted using a JEOL JEM-1400Plus (JEOL, Tokyo, Japan). The surface characteristics were further investigated using SEM (Philips-XL30 device, The Netherlands).

#### 2.9.5 XPS analysis

An X-ray photoelectron spectroscopy (XPS) was conducted using an Al Kα monochromatic excitation source on a VG Scientific ESCALAB220i-XL system. Following Shirley's background subtraction, the XPS data underwent quantitative analysis using the CasaXPS software. The XPS data were calibrated to a C1s standard binding energy of 284.5 eV, utilizing a PHI5000 VersaProbeIII analyzer.

#### 2.9.6 Raman spectroscopy (SERS) analysis of AgNPs

Raman spectra of the silver nanoparticles were obtained using a Renishaw Raman spectrophotometer with 633 nm Helium-Neon laser excitation at a power of 10 mW.

### 2.10 Antibacterial tests

#### 2.10.1 Bacteria strains preparation

The antibacterial activity was determined using the bacterial strains *Staphylococcus aureus* (ATCC 25923) and *Escherichia coli* (ATTCC 25922). All bacterial strains were cultivated using Mueller-Hinton broth (MHB) at 37 °C for 24 h.

#### 2.10.2 Antimicrobial activity using disc diffusion assay

The antimicrobial activities of the AgNPs, *C. gileadensis* extracts, and *Cg*-AgNPs against representatives of Gram-positive bacteria (*Staphylococcus aureus*) and Gram-negative bacteria (*Escherichia coli*) were assessed using the agar well diffusion method. The examined extracts were placed into sterile cork borer wells with a diameter of 7 mm and injected with 0.1 mL of the pre-culture from the tested bacterium (4 × 106 CFU/ mL) on each Muller Hinton agar plate containing 15 mL of the cooled medium. The diameter of the inhibitory zone was measured in millimeters after incubating for 24 h at a temperature of 37 °C. The bacteria *S. aureus* and *E. coli* were utilized as test subjects, while Tetracycline Hydrochloride and Amoxicillin were used as controls, respectively.

#### 2.10.3 Antibacterial activity using MIC and MBC

The MIC and MBC of AgNPs, *Cg*-extract, and *Cg*-AgNPs were determined. After the AgNP powder was exposed to UV light for 1 h to sterilize it, it was weighed while maintaining aseptic procedures. The antibacterial activity was measured using sterilized 2 mL 96-well platesSterilized. Mueller-Hinton agar (0.5 mL) was sterilized and poured into each of the 12 wells in each row. The concentration sequence of dilutions from 0.512 mL to 0.008 mL of extract was established by adding an additional 0.5 mL of a mixture of culture medium and serially diluted plant extract to wells 2–11. For this assay, well 1 served as a growth control, and well 12 was used as an antibiotic control. For the *S. aureus* and *E. coli* tests, 0.1 mg/mL of Tetracycline Hydrochloride and 0.1 mg/mL of Amoxicillin served as controls, respectively. The antibiotics were selected based on their frequent use as first-line treatments for the respective bacterial diseases. The MIC for *S. aureus* was determined to be 1.2 g/mL when tested with Tetracycline Hydrochloride, whereas the MIC for *E. coli* was 18 g/mL with Amoxicillin. At 37 °C, the deep wells were cultured for 24 h, resulting in some turbidity. The minimal inhibitory concentration (MIC) refers to the lowest antibacterial concentration preventing the observed growth in the tubes. Prior to and following placement in the incubator, the tubes were examined to confirm the MIC value.

The minimum bactericidal concentration (MBC) was determined by sub-culturing 100 µL of culture from each well of the micro-broth test for 24 h on MH agar plates in an incubator. An Extra 24-hour incubation was performed for MH plates. The minimum MBC endpoint is achieved when the antibacterial agent's lowest concentration destroys 99.9 % of bacterial cells. This was accomplished by examining agar plates before and after incubation to determine the presence or absence of bacteria.

### 2.11 Statistical analysis

After three replicates of each test, the results were reported as a mean ± standard deviation. The SPSS 22.0 (SPSS Inc., Chicago, USA) software program on Microsoft Windows 10 was used to conduct a one-way analysis of variance (ANOVA) utilizing Duncan's new multiple-range test. The tests were conduct to determine the statistically significant differences between means.

## 3 Results and discussion

### 3.1 Extraction yield (%)

The efficiency of techniques directly impacted extraction yield (%). Solubility, energy, time, and environmental and human impact determine extraction efficiency [30]. Environmental conditions like climate and soil, alongside human activities such as agricultural practices and extraction methods, directly influence the efficiency of bioactive compound extraction from plants. These factors affect both the quantity and quality of the compounds extracted. Therefore, it is crucial to develop a method for extracting bioactive phytochemicals from plant sources that is both cost-effective and environmentally friendly. In this study, bioactive chemicals in *C. gileadensis* dried leaves were extracted using four distinct extraction methods (traditional and non-traditional). The extract yield of *C. gileadensis* leaves prepared by MACE, USE, MICE, and Integrated US/MICE extraction techniques is presented in Table 1. It was determined that the extraction procedures impacted the extraction yield. The percentage of C. gileadensis extracts obtained after evaporation was determined [31].

**Table 1:** The yield% of various treatment extracts of *C. gileadensis*.

	MAC	US	MIC	US/MIC
DW(g)	0.8136^a^	0.4449^b^	0.4292^b^	0.4690^b^
%	20.34	11.12	10.73	11.73

MAC: maceration extraction; US: ultrasonic-assisted extraction; US/MIC: ultrasonic-microwave-assisted extraction; MIC: microwave-assisted extraction. DM: dry matter.

When comparing the yield (%) of *C. gileadensis* extracts, it was found that the maceration extract had the highest amount, while the MIC extract had the lowest percentage of *C. gileadensis* (Table 1).

Among the techniques studied, the maceration extract of C. gileadensis had the highest yield (20.34 %), while the extract of MICE had the lowest yield (10.73 %). The US/MICE and the USE yield were (11.73 and 11.12 %), respectively. The extract yield with the conventional method was 1.7-fold, 1.8-fold, and 1.9-fold higher than the yield obtained by US/MICE, USE, and MICE extract, respectively.

### 3.2 Effect of extraction technique on TPC

The examination of TPCs, which are associated with the antioxidant and functional properties of the extracts, was conducted based on the extraction methods used. Table 2, shows TPC (mg GAE/g) results for extraction methods. MACE, USE, MICE, and US/MICE *C. gileadensis* extracts contained 59.34 ± 0.007 to 94.47 ± 0.006 (mg GAE/g) of TPC. The impact of extraction techniques on TPC was significant, with US/MICE yielding the highest at 94.47. Subsequently, MICE recorded 83.26, USE 68.50, and MACE 59.34 mg GAE/g. The TPC yield was obtained by US/MICE, MICE, and USE individually. Notably, the US/MICE technique involved only half the amount of acoustic wave exposure compared to the USE method. Comparing our findings to those of other researchers, such as Kamgaing et al. [32], we found that our findings were more positive. Their study found that TPC (mg GAE/g) was extracted using maceration, a traditional method. For some of the *Burseraceae* family the TPC was found to be 5.53 in the aqueous extract of Dacryodes edulis (5.53). The TPC values obtained from the Commiphora leptophloeos extracts were significantly higher than those obtained from other solvents with different polarities (chloroform, ethyl acetate, methanol, and water) used for soaking extraction. The analysis of total phenolic content showed that the aqueous (33.64) and methanolic (20.3) extracts exhibited the highest phenolic content [33]. Furthermore, the TPC results of US/MICE were superior to those of conventional extraction from *Commiphora africana* (65.00) [34]. However, the TPC in the methanolic extracts of Canarium tramdenum was higher (112.14) than the TPC results obtained in this study [35].

**Table 2:** TPC, % DPPH (IC_50_ and EC_50_) values of *C. gileadensis* treatments.

EM	TPC mg GAE/g DM	IC_50_ (μg/mL)	EC_50_ (μg/mL)
US/MIC	94.47 ± 0.006	48.25	6.48
MIC	83.25 ± 0.004	41.82	6.55
US	68.50 ± 0.005	25.39	6.66
MAC	59.34 ± 0.007	21.60	7.21
TBHQ	ND	90.27	4.36

EM: extraction methods; MAC: maceration extraction; US: ultrasonic-assisted extraction;

US/MIC: Ultrasonic- microwave-assisted extraction; MIC: microwave-assisted extraction.

The IC_50_ and EC_50_ were calculated as μg/mL. ND: not determined.

The variation in the TPC values can be attributed to the solvent and extraction time conditions significantly impacting the TPC. Nevertheless, high-wave exposure causes a temperature rise, a substantial factor in degrading phenolic materials. There are several drawbacks associated with this process. These include the deterioration of phenolic compounds initially released at lower temperatures, the breakdown of any remaining phenolics in the plant matrix, and increased solvent loss due to vaporization and oxidation processes [35], [36].

Ultrasonic/microwave-assisted strategies for extracting bioactive compounds from plant materials offer a significant advantage over conventional extraction methods. The effects of microwave and ultrasound on the samples proved that the results had been obtained. After exposure to high-intensity radiation, molecules gain more kinetic energy and transfer mass more quickly, increasing the amount of active ingredients extracted [37], [38]. However, prolonged microwave and/or ultrasonic irradiation of biological materials might have adverse effects such as phenolic compounds or flavonoids oxidation, degradation and/or polymerization [39], [40].

### 3.3 Antioxidant activity (DPPH assay) of *C. Gileadensis*

**Fig. 1:**
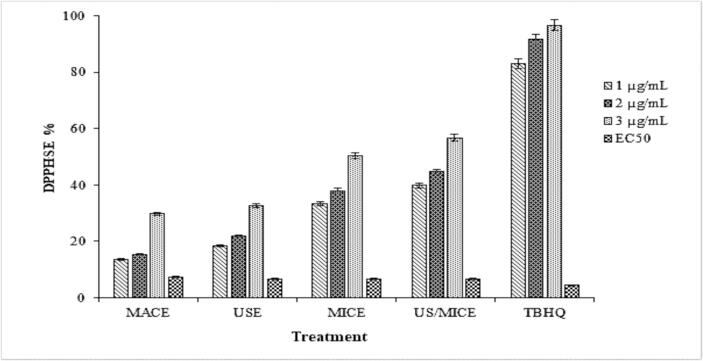
Antioxidant capacity (DPPH scavenging effect) of *C. gileadensis* extract.

Antioxidant chemicals and extracts from the Burseraceae family have been tested using DPPH, a relatively stable organic radical [41], [42]. To evaluate the antioxidant activity of *C. gileadensis*, different extraction techniques were measured and compared with their DPPH radical scavenging activities. Fig. 1, demonstrates a concurrence between the antioxidant activity values obtained from the ABTS assay and those obtained from the DPPH assay.

The US/MICE treatment of the *C. gileadensis* sample resulted in the highest DPPH (%) values of 56.69 %, 44.71 %, and 39.80 % at concentrations of 3, 2, and 1 µg/mL, respectively. In contrast, the comparable treatment (TBHQ) showed significant differences (*p < 0.05*) with higher DPPH (%) values: 96.67 %, 91.64 %, and 83.05 % at concentrations of 3, 2, and 1 µg/mL, respectively. The EC_50_ value obtained by the TBHQ sample (4.36) was lower than the obtained by the US/MICE treatment (6.48 µg/mL).

Regarding the analysis of DPPH radicals (Fig. 1), extracts obtained through MACE at 40 °C exhibited higher antioxidant activity (83.5 mg gallic acid/g dm) in comparison to USE (68.5 mg gallic acid/g dm). Furthermore, the extract obtained through microwave extraction exhibited a more significant antioxidant activity compared to maceration (59.3 mg gallic acid/g dm). Consequently, the exposure of the extract to microwaves was more advantageous to its antioxidant activity. A potential explanation is that the USE extract may have decreased activity due to some ultrasound-related effect. However, MICE offers the advantage of reduced solvent use and a shorter extraction time compared to the more time-consuming MACE extraction method.

### 3.4 ABTS scavenging effect of *C. Gileadensis*

**Fig. 2:**
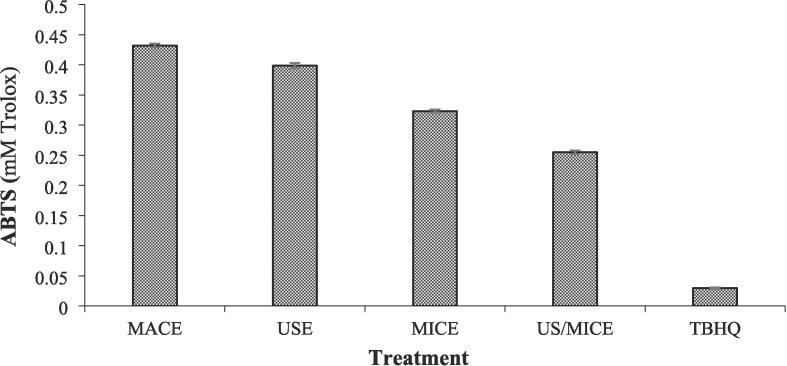
Antioxidant capacity (ABTS scavenging effect) of *C. gileadensis* extract.

The antioxidant capacity is frequently investigated using the DPPH and ABTS free radical-scavenging activity assays due to their simplicity and accuracy simplicity and accuracy [27], [43]. Fig. 2 depicts the results of ABTS (as mM Trolox), which were as follows: TBHQ (0.030), US/MICE (0.255), MICE (0.323), USE (0.399), and MACE (0.432). A significant correlation (expressed as ABTS value in percentage) was observed between TBHQ and all treatments of *C. gileadensis*. In addition all treatments of *C. gileadensis*. Besides, all treatments of the *C. gileadensis* sample under study showed significant differences, with the US/MICE treatment yielding the highest ABTS value.

This study found that extracts of *C. gileadensis* obtained using the MICE, USE, and MACE exhibited comparable levels of antioxidant activity. Statistically significant differences were observed when comparing the US/MICE extracts to those obtained using the other three methods. This could be due to the partial decomposition of specific compounds with antioxidant properties following prolonged treatment and intense heating. While there were slight differences in antioxidant activity among the tested extracts, the overall findings were consistent, suggesting that the different extraction methods employed did not significantly alter the phenolic content of the extracts. The essay's DPPH scavenging effect is based on the presence of natural TPC and their ability to donate a hydrogen ion to convert DPPH radicals from DPPH• (free radical) to DPPH-H (non-radical) [44], [45]. In the current study, the samples extracted from *C. gileadensis* using MICE exhibited higher TPC and antioxidant activity than USE and MACE.

### 3.5 Characterization

#### 3.5.1 X-ray diffraction (XRD)

**Fig. 3:**
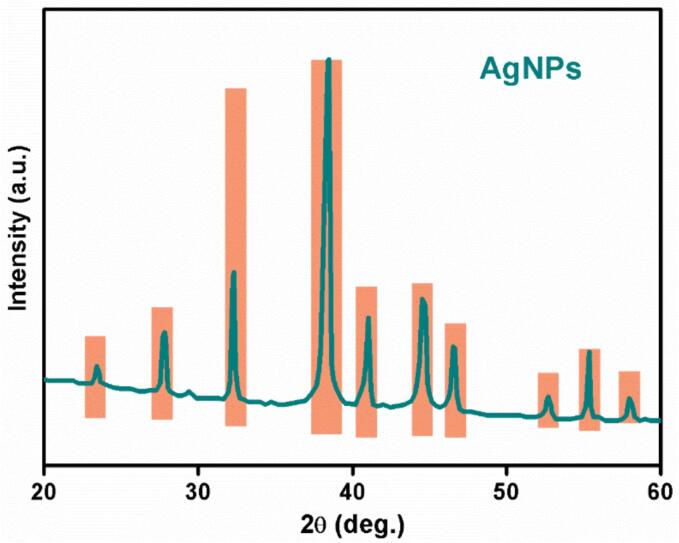
XRD of AgNPs of *C. gileadensis*..

The X-ray diffraction (XRD) pattern of the AgNPs is depicted in Fig. 3. The peaks were indexed, and Miller indices were used to identify the crystalline phases present. The observed reflections corresponded to AgNPs with face-centered cubic symmetry [46], [47]. The high-intensity peaks indicated that the AgNPs were highly crystalline [48]. The diffractogram showed peaks at 23.46°, 27.81°, 38.36°, 44.56°, 46.53°, 55.10°, and 57.60°. These peaks corresponded to face-centered cubic metallic silver (JCPDS 76–1393) [49]. This confirms that the main component of the nanoparticles was Ag metal.

#### 3.5.2 Uv–vis of AgNPs

**Fig. 4:**
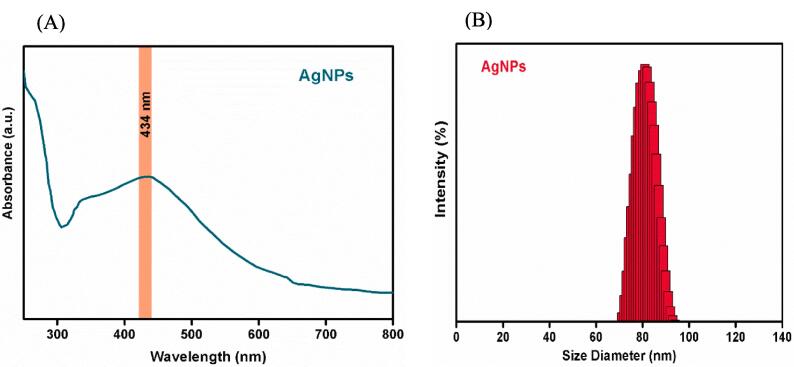
(A) UV–visible absorption spectra, and (B) Particle size analysis of AgNPs of *C. gileadensis*.

The UV–Vis spectrum exhibited the maximum absorbance at 434 nm (Fig. 4 A and B), corresponding to the characteristic wavelength of AgNPs. This resonance peak, approximately 434 nm, has been extensively documented in various studies on AgNPs [50].

#### 3.5.3 DLS analysis

The average diameters of the AgNPs were found to be 92 ± 10.2 nm (with PDI = 0.35 ± 0.02).

#### 3.5.4 XPS studies

**Fig. 5:**
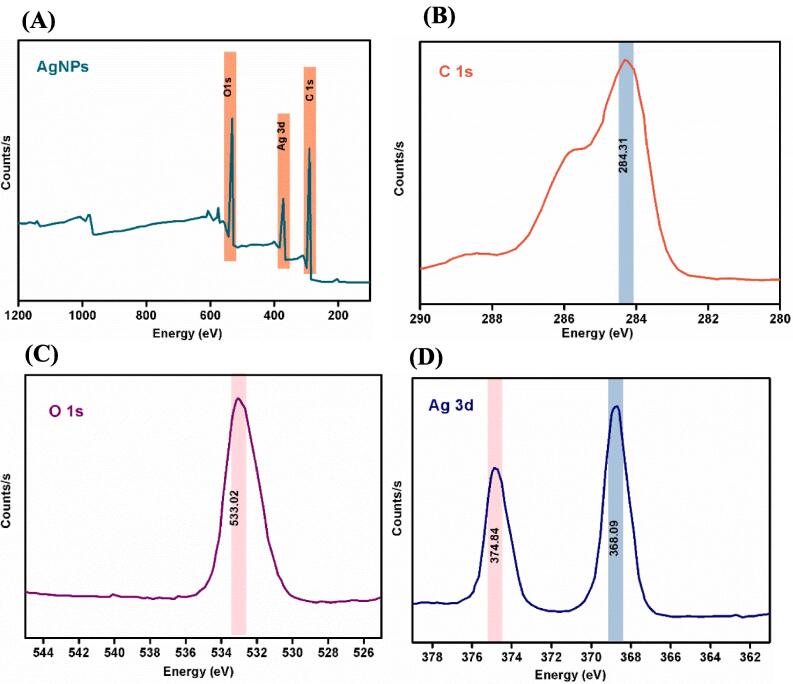
A-D. XPS studies of Ag. NPs. X-ray photoelectron spectroscopy (XPS) was employed to characterize the synthesized materials (Fig. 5A). The smaller energy Ag 3d3/2 peak and the higher energy Ag 3d5/2 peak are illustrated in Fig. 5B. C1s peaks with binding energies of 284.04–284.31 eV (Fig. 5C) revealed the presence of adventitious carbon at lower energy and carbonate carbon at higher energy. The peak at 533.02–533.21 eV (Fig. 5D) indicated the presence of adsorbed oxygen species.

The synthesized materials were characterized using X-ray photoelectron spectroscopy (XPS) (Fig. 5A). Low-resolution scans with a 1 eV step energy were conducted over a binding energy range of 0–1361 eV. Resolving Ag ions using XPS is difficult because Ag metal and silver oxide have similar binding energies. The Ag 3d5/2 peak, representing Ag, Ag2O, and AgO species, was observed at 368.09–374.84 eV. Fig. 5B depicts the Ag 3d3/2 peak with lower energy and the Ag 3d5/2 peak with higher energy. The C1s peaks with binding energies of 284.04–284.31 eV (Fig. 5C) revealed the presence of adventitious carbon at lower energy and carbonate carbon at higher energy. The presence of adsorbed oxygen species is indicated by the peak observed at 533.02–533.21 eV in Fig. 5D.

#### 3.5.5 Surface-enhanced Raman spectroscopy (SERS) analysis

**Fig. 6:**
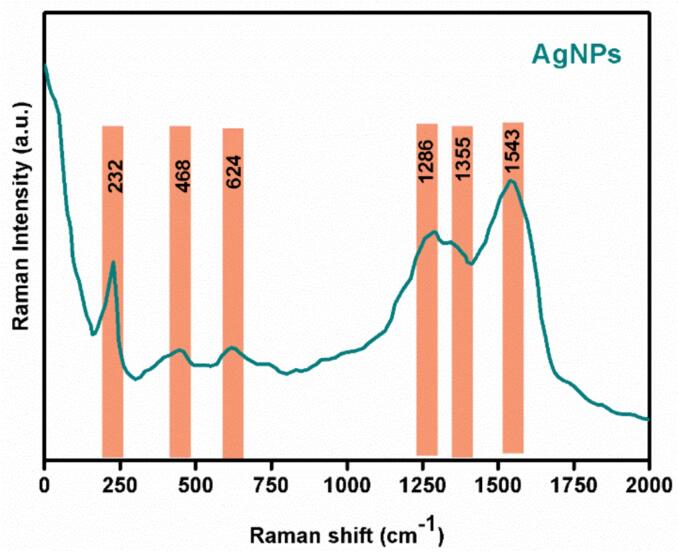
Raman scattering of AgNPs.

Surface-enhanced Raman spectroscopy (SERS) is a well-established method used extensively to characterize metal nanoparticles. Creighton et al. first demonstrated the capability of colloidal AgNPs in aqueous media to enhance Raman signals [51]. With advancements in plasmonic research and an enriched understanding, SERS has evolved into a valuable analytical method for sensitive chemical analysis and interfacial studies. It is widely recognized that optimal SERS enhancement is achieved using rough silver surfaces, such as AgNPs in various shapes. Over the past decade, extensive research has been conducted to control size and shape, as these factors play a crucial role in adjusting nanomaterial properties. Fig. 6 displays the Raman spectrum of AgNPs, exhibiting vibrational modes at 232, 468, 624, 1286, 1355, and 1543 cm-1. The AgNPs were synthesized using *C. gileadensis* extract, which also acted as a surfactant. The extract contained organic components such as carboxylic and hydroxyl groups. In the Raman spectrum, the peak at 232 cm-1 corresponds to Ag-O stretching [52], while the 468 and 624 cm-1 peaks arise from C-N-C and C-S-C stretching vibrations [53]. The other bands at 1286, 1355, and 1543 cm-1 originate from carboxylic symmetric and anti-symmetric C = O stretching vibrations [54], [55].

#### 3.5.6 Surface morphology (TEM and SEM study)

**Fig. 7:**
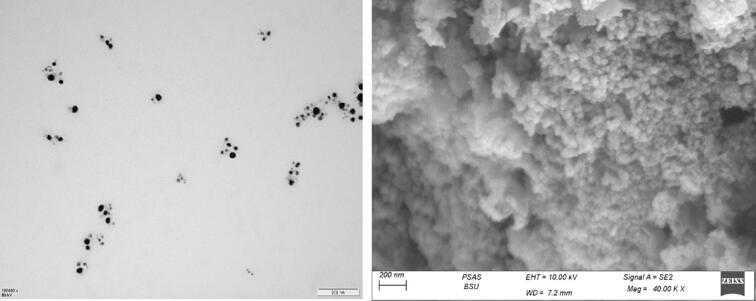
Characterization of the size and structure of silver-*Cg* nanoparticles by using **(A)** TEM analysis, and **(B)** SEM photographs.

TEM analysis of the reaction solution was conducted to investigate the impact of synthesis conditions on the size, shape, size distribution, and crystalline structure of AgNPs. Analysis of a biosynthetic reaction under a microscope confirmed the synthesis of AgNPs. The TEM images of biosynthesized nanoparticles are shown as examples in Fig. 7. As shown in the micrograph, agNPs are typically characterized by their distinctive characteristics, exhibiting nearly spherical shapes and minimal or no particle aggregation. The findings revealed that the synthesized Cg-AgNP exhibited a spherical shape, with nanoparticle diameters ranging from 7.7 to 42.9 nm (Fig. 7).

TEM assays are widely used to characterize and validate the size and morphology of AgNPs and/or AgNPs synthesized through green methods [56], [57], [58]. As shown in Fig. 7, scanning electron microscopy (SEM) was employed to examine the morphology of the synthesized Ag NPs. SEM analysis revealed that the nanoparticles exhibited a spherical shape with a non-uniform distribution. The average size of the particles was determined to be 30 ± 5 nm.

### 3.6 Chemical characterization of *Cg* extract using UHPLC‐Qtof‐MS/MS

*C. gileadensis* is known as a “storehouse” of bioactive compounds, such as polyphenolics and aliphatic alcohol glycosides, that have significant potential for pharmaceutical applications [59].

**Fig. 8:**
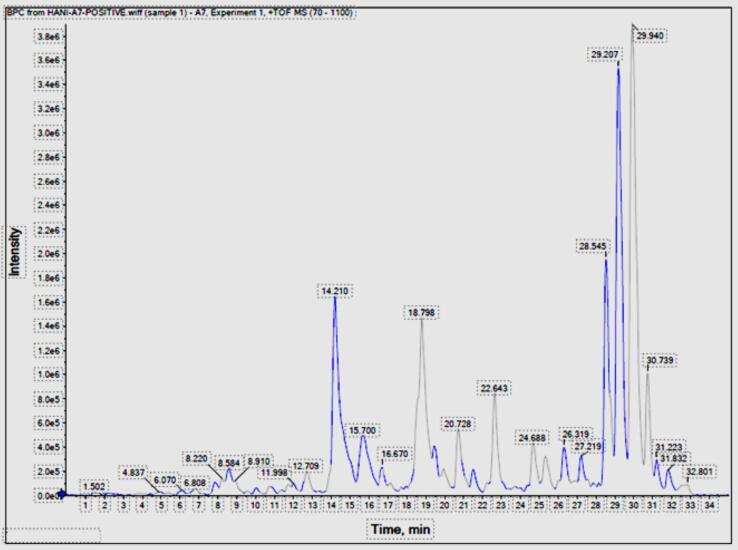
UHPLC‐qTOF‐MS/MS base peak chromatogram of *C. gileadensis* extract in positive ionization mode.

In this study, the US/MIC extract was used to explore the technical abilities of extracting secondary metabolites from *C. gileadensis*. There is currently no extensive research on identifying and characterizing the phytoconstituents in different parts of C. gileadensis. UHPLC-qTOF-MS/MS separates the components, accurate mass, and MS/MS data, enabling identification and characterization of the components. Therefore, the UHPLC-qTOF-MS/MS analytical platform is used in ESI-positive mode. Comprehensive metabolite profiling was performed, and 64 chromatographic peaks were detected, out of which 58 were annotated (Fig. 8).

**Fig. 9:**
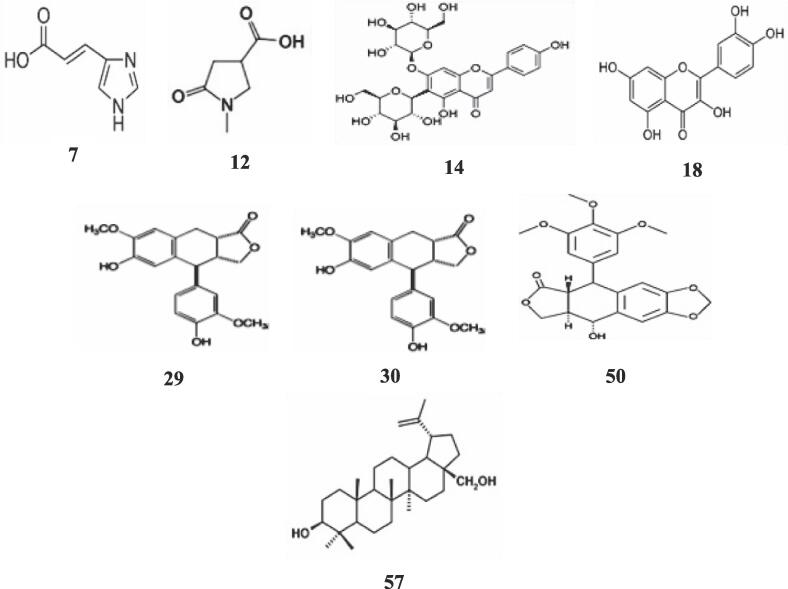
Representative structures of major metabolites identified in *C. gileadensis* extract.

The identified metabolites in the resulting base peak chromatograms (BPCs) belonged to various classes, including 12 organic compounds and their derivatives, eight polyphenols (flavonoids (5) and lignans (3), seven amines and amides, seven terpenes, six fatty acid derivatives, four peptides, and amino acid derivatives, four steroid and sterols derivatives, two nucleotide bases, two carbohydrate derivatives, two heterocyclic compounds, two vitamins, one alkaloid, and others seven compounds. The chemical compositions of some chosen metabolites identified in the *C. gileadensis* extract are shown in Fig. 9. This is the first study to utilize high-resolution UHPLC-MS/MS to characterize the metabolites of *Cg*-US/MIC extract. In the current study, we detail the abundant classes.

#### 3.6.1 Organic compounds and their derivatives

The extract of *C. gileadensis* contained a high concentration of organic acids. The majority of compounds identified in *C. gileadensis* are organic compounds and their derivatives. A total of 12 organic acid peaks were identified in the leaf part of *C. gileadensis*. Peaks 3, 9, 12, 13, 17, 19, 20, 27, 39, 44, 47, and 56 were positively identified by comparing their RT and MS with those of authentic reference. The peaks were identified and characterized based on their exact masses and MS/MS as 2,6-Deoxyfructosazine, Diethyl 2-[(*tert*-butoxycarbonyl) amino] malonate, Methyl-5-oxo-3-pyrrolidinecarboxylic acid, Diethyl 1,2,6-trimethyl-4-(5-methyl-2-thienyl)-1,4-dihydro-3,5-pyridinedicarboxylate, 1-(4-Nitrophenyl)-4-(3,4,5- trimethoxy benzoyl) piperazine, (5E)-1-Benzyl-5-(1-([2-(diethylamino) ethyl] amino) propylidene)-2,4,6 (1H,3H,5H)-pyrimidinetrione, Glutaric acid, tridec-2-yn-1-yl 3-nitrobenzyl ester, Galbelgin, Sebacic acid, octyl 1-phenylpropyl ester, Hydroxy-3′,19-dimethoxy-4′,5′-didehydro-5′,6′,7,8-tetrahydro-beta, beta-caroten-8-one, Dimethylmalonic acid, 4-acetylphenyl ethyl ester, (2E)-1-[4-(Methylsulfanyl) phenyl]-3-[4-(octadecyloxy) phenyl]-2-propen-1-one, respectively. 2,6-Deoxyfructosazine exerts physiological and pharmacological effects on the human body, including the regulation of blood sugar levels, inhibition of cancer cells, and treatment of diabetes [60].

#### 3.6.2 Polyphenol compounds

Phenolic compounds can be classified into several families based on their structure, consisting of aromatic rings with one or more hydroxyl groups. The primary phenolic constituents found in Cg extracts at different concentrations are flavonoids and lignans.

##### 3.6.2.1 Flavonoids

Flavonoids are among the most significant phytochemicals of phenolic compounds, found in various plants, fruits, and vegetables. They are also known for their wide range of health advantages [61]. Flavonoids exhibit a wide array of beneficial activities, including antioxidant, anticancer, antiviral, anti-inflammatory, neuroprotective, and cardioprotective effects. Their chemical composition, particularly the hydroxy groups, impacts their bioavailability and biological activity [62], [63]. The fundamental structure of flavonoids consists of two benzene rings (A-B) connected by a three-carbon pyran ring. Their antioxidant capacity is determined by the position of the catechol B-ring and the number of hydroxy groups on the *B*-ring [64], [65].

Flavonoids are a vital group of phenolic compounds widely distributed in *Cg*. A total of 5 flavonoids were identified and characterized. Among them, 19 compounds (14, 18, 32, 41, 43) were positively identified by comparing their RT, MS, and MS/MS patterns with those of authentic reference. Peak 43 was identified as isoflavone at *m*/*z* 385. The UHPLC‐Qtof‐MS/MS analysis of *Cg* detected two distinct peaks corresponding to saponarin and quercetin, with retention times of approximately 6.92 and 8.90 min, respectively. Saponarin has been studied for its potential in various therapeutic areas, including its role as an antioxidant, hepatoprotectant, anti-inflammatory, anti-allergic agent, skin protector, and anti-diabetic compound[66]. Quercetin, a type of flavonoid, possesses unique biological properties and ameliorates aging processes and chronic diseases in humans. Many studies have been conducted on its potential antiviral effects [67]. The structures of saponarin (14) and quercetin (18) are shown in Fig. 9.

##### 3.6.2.2 Lignans

Lignans are a group of natural chemicals produced by various plant species. Recent research has revealed the biological and pharmacological properties of lignans, encompassing anti-inflammatory, immunosuppressive, cardiovascular, anticancer, antioxidant, and antiviral activities, have recently come to light in research. Several plants used in Eastern medicine contain these compounds [68], [69]. Cinnamic acid, cinnamyl alcohol, propenyl benzene, and allyl benzene are a few examples of monomers constituting the fundamental structure of lignans, which comprise two or more phenylpropanoid units [70].

Three lignans were identified and characterized in Cg extracts. They included podophyllotoxin at *m*/*z* 415.1370, anthricin at *m*/*z* 399.1419, and β-conidendrin at *m*/*z* 385.1631. Podophyllotoxin is a non-alkaloid toxin lignan derived from various plants' roots and rhizomes. It possesses notable antiviral and antineoplastic properties. Etoposide and teniposide are semisynthetic podophyllotoxin derivatives, both therapeutically significant anticancer medications. Both medications affect DNA and have been found to influence topoisomerase II [71]. Conversely, anthricin (deoxypodophyllotoxin) is one of the main lignans and has a wide range of bioactivities, including antiproliferative, anti-platelet aggregation, anticancer, antiviral, and anti-inflammatory effects [72].

#### 3.6.3 Terpenes

UHPLC‐Qtof‐MS/MS was utilized to analyze the components of Cg leaves, and seven compounds were identified in the class of terpenes. Compound 23, at a retention time of 11.00, showed molecular ions [M + H]^+^ at *m*/*z* 493.2786, corresponding to the molecular formula of C_27_H_40_O_8_. Compound 23 was tentatively identified as sordarin. Sordarin is one of the few antifungal medicines specifically targeting the fungus's translational machinery. Sordarin acts on eukaryotic translation elongation factor 2 to halt protein synthesis during the elongation phase of the translational cycle [73]. The majority of terpenes exhibit fragments with *m*/*z* values of 347.1843 [C_20_H_26_O_5_]^+^, 433.1845 [C_27_H_40_O_8_]^+^, 499.3763 [C_32_H_50_O_4_]^+^, 443.3868 [C_30_H_50_O_2_]^+^, 457.3661 [C_30_H_48_O_3_]^+^ and 441.3704 [C_30_H_48_O_2_]^+^.

#### 3.6.4 Amides and amines

Amide bonds are present in the majority of organic compounds and biomolecules, such as peptides, proteins, DNA, and RNA. The ability of amide bonds to create resonant structures distinguishes them from other types of bonding. Consequently, they exhibit high stability and assume specific three-dimensional structures responsible for their activities [74]. Amines are crucial nitrogen-containing molecules in plant, microbial, and animal cells [75].

Using UHPLC‐Qtof‐MS/MS analysis, we identified 64 metabolites, specifically seven peaks characterized as amides and amines. Amides and amines, namely, N-(3-methylphenyl)-2-([5-(3-toluidino)-1,3,4-thiadiazol-2-yl]sulfanyl)acetamide (1), 3-phenyl-1-[4-(4-phenyl-piperazin-1-yl)-butyl]-1H-indazol-5-ylamine (21), 2-(2,4-ditert- pentyl phenoxy) −N-(2-hydroxyphenyl) butanamide (24), N-[5-(phenoxymethyl)-4,5-dihydro-1,3-oxazol-2-yl]-N,N'-diphenyldicarbonimidic diamide (25), 17-phenyl trinor prostaglandin F2α cyclohexylamine (30), N-(4-([amino (imino) methyl]amino)-1-[(4-nitroanilino) carbonyl]butyl) benzamide (37), N-(5-(diethylamino)-2-[(E)-(2,4-dinitrophenyl)diazenyl]-4-methoxyphenyl) acetamide (49) were identified in the *Cg* extract.

#### 3.6.5 Fatty acid derivatives

Fatty acid derivatives are essential components in plants since they possess some biological activities that are closely associated with biosynthesis. The UPLC-QTOF-MS analyses identified fatty acid molecules. A total of 6 fatty acid derivatives were tentatively identified. The observed peaks in the positive ion mode correspond to fatty acid derivatives that display [M + H] + ions. Peak 6 (*m*/*z* 146.0919) was tentatively identified as 8-aminocaprylic acid (C8H17NO2). This compound belongs to a series of amino-substituted carboxylic acids capable of forming hydrogen bonds. Based on a report on a novel tetragonal phase of γ-aminobutyric acid, it has been observed that this phase hinders the ability of nerve cells to receive, complete, or send chemical messages to other nerve cells. This plays a significant role in regulating anxiety, stress, and fear [76]. The findings provide new insights into the beneficial phytochemicals present in *C. gileadensis*.

### 3.7 In vitro antimicrobial activity of green synthesized *Cg-*AgNPs

The test aimed to determine the antibacterial activity of the green synthesized AgNPs. This assay is commonly used to determine the size and morphology of AgNPs, as well as to confirm their synthesis through green methods. According to the TEM image, the Cg-AgNPs are spherical, with an average particle size of 25.3 nm.

The antibacterial efficacy of *Cg*-extract, *Cg*-AgNPs, and AgNPs were examined using *E. coli* (ATTCC 25922), a type of Gram-negative pathogenic bacteria, and *S. aureus* (ATCC 25923), a Gram-positive pathogenic bacteria. The results for the disk diffusion assay, MIC, and MBC of the extracts are depicted in Table 3, Table 4.

**Table 3:** Assaying the MIC and MBC evaluation of AgNPs, *Cg*-extract and *Cg*-AgNPs.

Sample	Inhibition zones (mm)	
	Gram (^+^ve) pathogenic bacteria	Gram (^−^ve) pathogenic bacteria
	*S. aureus*	*E. coli*
*Cg-*extract	14 ± 1	−
*Cg*-AgNPs	16 ± 2	14 ± 1
AgNPs	14 ± 2	14 ± 0.4
Control	12 ± 3	16 ± 2

–; no inhibition zone, Data are means of three replicates (n = 3).

**Table 4:** Antimicrobial screening test of AgNPs, *Cg*-extract and *Cg*-AgNPs against some bacterial strains.

Bacterial strain	Control *mg /ml*		AgNPs *mg/ml*		*Cg-*extract *mg/ml*		*Cg*-AgNPs *mg/ml*	
	MIC	MBC	MIC	MBC	MIC	MBC	MIC	MBC
*E. coli*	0.018	0.018	6.8	6.8	7	5	5	3
*S. aureus*	0.0012	0.0012	1.75	1.75	4	2	1.75	0.75

MIC; Minimum inhibitory concentration, MBC; Minimum bactericidal concentration. *S. aureus*; *Staphylococcus aureus* ATCC 25923, *E. coli*; *Escherichia coli* ATCC 25923.

The disk diffusion assay demonstrated that, apart from the *Cg*-extract with *E. coli*, the extracts exhibited antibacterial activity that effectively suppressed the growth of both Gram-negative and Gram-positive pathogenic bacteria. This was evident from the presence of a distinct, clear zone surrounding the *Cg*-extract, *Cg*-AgNPs, and AgNPs. The results demonstrated the efficacy of the extracts in suppressing the growth of the studied pathogenic bacteria. The average inhibition diameter was 14 ± 2 mm in *S. aureus* and 14 ± 1 mm in *E. coli*, as shown in Table 3.

The findings demonstrated the efficacy of AgNPs in suppressing the growth of the pathogenic bacteria under study. The average diameter of inhibition was measured to be 14 ± 2 mm in *S. aureus* and 14 ± 1 mm in *E. coli*, as indicated in Table 3. These results are in agreement with a previous study conducted by Al-johny (2019). They demonstrated the feasibility of using AgNPs to inhibit the growth of *E. coli* and *S. aureus* and their ability to change bacterial cell membranes by interact ing with sulfur in the bacterial cell wall [77]. This enhances the membrane's permeability, ultimately leading to the demise of the bacterial cell. The addition of *Cg*-extract to the Mueller-Hinton culture medium using the diffusion method resulted in an inhibition diameter of 14 ± 1 mm in *S. aureus* bacteria. This finding was more significant than that of the study conducted by Al-Hazmi (2020) for the methanolic extract of C. gileadensis, which indicated that the zone of inhibition was 7 mm [78]. This result is mainly due to the mode of action of the plant extract proposed by Strobel et al. (2004). In this mechanism, the active component of the extract may be complex with some bacterial cell walls, destroying the bacterial cell wall, or complex with its chromosomal DNA [79]. However, Danial and Majrashi (2016) reported the absence of inhibition in *E. coli* bacteria. Additionally, it was found that the antimicrobial effectiveness of *Cg*-extract against Gram-negative bacteria is comparatively reduced and less responsive to the extracts compared to its effectiveness against Gram-positive bacteria [80]. The results of the *Cg*-AgNPs demonstrated a significantly greater inhibitory effect of 16 ± 1 compared to both AgNPs and *Cg*-extract extracts. The percentage increase in inhibition was 14.29 % when compared to the control, whereas the control exhibited a 33.33 % increase in *S. aureus* bacteria. Both AgNPs and *Cg*-AgNPs extracts in *E. coli* exhibited a comparable inhibition of approximately 14 mm.

This finding agrees with many studies that demonstrate the ability of AgNPs to inhibit bacteria when combined with plants. The use of AgNPs results in greater inhibition compared to plants alone, indicating the enhanced efficiency of nanoparticles in augmenting the inhibitory properties of plant compounds against microorganisms. The results are consistent with many other studies demonstrating the efficiency of nano-silver enriched with plants in inhibiting microorganisms that produce toxins, such as *E. coli* and *S. aureus* [81], [82], [83], [84]. These results demonstrated the possibility of increasing the inhibitory characteristic of the *Cg*-extract after synthesis of *Cg*-mediated AgNPs to inhibit the growth of bacteria resistant to antibiotics. Therefore, there is potential to use *Cg*-AgNPs as an alternative to antibiotics in infections caused by pathological bacteria under study, especially S. aureus.

The disk diffusion assay served as an initial investigation to screen for antibacterial activity. Therefore, a subsequent assessment was necessary to determine the antibacterial activity of the extracts using the MIC and MBC values. The MIC of an antibacterial agent was determined using a process of serial dilution. Table 4 illustrates that the MIC values of the extracts against the bacteria *E. coli* varied between 5 and 7 mg/mL. The MIC value of *Cg*-AgNPs was 5 mg/mL, while AgNPs had a MIC value of 6.8 mg/mL, and *Cg*-extract had a MIC value of 7 mg/mL. For *S. aureus*, the MIC ranged from 1.75 to 4 mg/mL. For *Cg*-AgNPs and AgNPs, it was 1.75 mg/mL, while *Cg*-extract showed an MIC value of 4 mg/mL. MBC is the lowest concentration of antibacterial agent to kill the bacteria (there was no growth on the agar plate). In the study, the MBC of *S. aureus* for *Cg*-AgNPs, AgNPs, and *Cg*-extract were 0.75, 1.75, and 2 mg/mL, respectively, while the MBC of *E. coli* were 3, 5, and 6.8 mg/mL, respectively. *S. aureus* was observed to be sensitive to *Cg*-AgNPs and AgNPs, with MIC values of 1.75 mg/mL and MBC values of 0.75 and 1.75 mg/mL, respectively. *E. coli* was less sensitive with MIC and MBC values compared to *S. aureus* (Table 4). This may be attributed to the positive charges of AgNPs trapped and blocked by lipopolysaccharide, thereby reducing the susceptibility of *E. coli* to AgNPs.

AgNPs have been extensively utilized for the prevention and treatment of different diseases for many years due to their potent biocidal impact against microorganisms [85]. Recently, non-hazardous AgNPs were successfully synthesized using a simple and cost-effective approach. These AgNPs were then assessed as novel antimicrobial agents.

The current study evaluated extracts for their potential as an antibacterial agent against selected Gram-negative and Gram-positive bacteria cultured in a petri dish. It was observed that *Cg*-AgNPs, *Cg-*extract, and AgNPs significantly increased the inhibition of the tested Gram-positive bacteria more than the control bacteria compared to Gram-negative bacteria. This finding can be ascribed to the composition of the cell walls of Gram-negative bacteria, as Gram-negative bacteria possess a unique cell wall structure distinct from Gram-positive bacteria. The structure consists of a cytoplasmic membrane, a thin peptidoglycan layer, and an outer membrane containing lipopolysaccharides. The periplasmic space, also called the periplasm, is located between the cytoplasmic and external membranes. It contains a loose network of peptidoglycan chains known as the peptidoglycan layer [83], [84].

According to Sheng et al. (2022), smaller AgNPs have a greater surface area than larger ones, which can make them more toxic to bacteria and have more potent bactericidal effects [86]. Bharti et al. (2021) found that the size and shape of AgNPs may influence their bactericidal efficacy. They also concluded that the surface charge of AgNPs significantly affects their interactions with bacterial cell surfaces. Therefore, there is considerable potential for using anisotropic AgNPs as efficient antimicrobials in medical applications [87]. Several researchers have investigated the antibacterial properties of plant-mediated AgNPs against pathogenic bacteria, and it has been demonstrated that plant-mediated-AgNPs are efficient against a wide range of pathogenic bacteria, such as *E. coli* [88], [89], *Pseudomonas aeruginosa* [90], *S. aureus* [89], [91], and *Bacillus subtilis* [88].

The exact mechanisms by which AgNPs eliminate bacteria remain unknown. However, some studies have hypothesized that AgNPs may affect bacteria due to their ability to adhere to and penetrate cells, enabling AgNPs to infiltrate and damage the cell membrane, thereby releasing intracellular contents [92], [93]. Silver ions are released inside the cell to damage the respiratory chain, leading to oxidative stress and increasing the formation of reactive oxygen species (ROS) [94]. Additionally, silver ion cytotoxicity involves deactivating proteins inside the cell [95].

Comparing AgNPs to chemical antibacterial agents is beneficial due to the prevalence of antimicrobial resistance associated with chemical antimicrobial agents. Several microorganisms have developed resistance to chemical antimicrobial agents through various mechanisms, severely limiting their utility in medical applications. Plant-mediated AgNPs can serve as an alternative strategy for combating bacterial resistance. Unlike traditional chemical antibacterial agents, bacteria are less likely to develop resistance to metal nanoparticles. AgNPs possess antibacterial properties and can exert their effects through various mechanisms.

## 4 Conclusion

In conclusion, the evolution of multi-resistant pathogens poses an obstacle to healthcare systems. Therefore, there is an urgent need to find alternative therapies, such as NPs, to develop safe and effective alternatives to pathogenic bacteria due to antimicrobial resistance. As a result, an eco-friendly methodology was used in this study, referred to as “green synthesis” using *C. gileadensis* leaf extract to produce AgNPs. Prior to the use of *C. gileadensis* leaf extract mediated by AgNPs, various methods, including microwave, ultrasonic, integrated ultrasonic/microwave-assisted, and maceration extraction, were utilized to enhance the extraction of polyphenols from *C. gileadensis* leaves. The extracts derived from *C. gileadensis* exhibited promising bioactivity. Therefore, they can be considered potential plant-based bioactive agents for anti-free radicals and antimicrobial agents. An extensive extract analysis was conducted using UPLC-qTOF-MS/MS to identify the active compounds present. A total of 64 chromatographic peaks were detected, out of which 60 were annotated. Polyphenols (flavonoids, lignans), organic compounds and their derivatives, amides and amines, terpenes, and fatty acid derivatives were identified as the most common classes of metabolites. The TEM analysis revealed that the synthesized material exhibited primarily spherical morphology with minimal aggregation. The results of the present study showed that the integral *Cg*-AgNPs exhibited potent antibacterial activity against pathogenic bacteria.

## CRediT authorship contribution statement

**Hani Ahmed:** Writing – original draft, Validation, Methodology, Investigation, Formal analysis, Data curation, Conceptualization. **Mohamed Y. Zaky:** Resources, Investigation. **Marwan M. A. Rashed:** Writing – review & editing, Supervision, Methodology. **Marwan Almoiliqy:** Formal analysis. **Sam Al-Dalali:** Formal analysis. **Zienab E. Eldin:** Formal analysis. **Mohanad Bashari:** Data curation, Conceptualization. **Ahmad Cheikhyoussef:** Writing – review & editing, Investigation. **Sulaiman A. Alsalamah:** Writing – review & editing, Funding acquisition. **Mohammed Ibrahim Alghonaim:** Writing – review & editing, Funding acquisition. **Abdulrahman M Alhudhaibi:** Writing – review & editing, Funding acquisition. **Jinpeng Wang:** Writing – review & editing, Funding acquisition. **Li-Ping Jiang:** Resources.

## Declaration of competing interest

The authors declare that they have no known competing financial interests or personal relationships that could have appeared to influence the work reported in this paper.
